# K-Ras4A Plays a More Significant Role than K-Ras4B in Ductal Carcinoma of Breast

**DOI:** 10.52547/ibj.3857

**Published:** 2023-01-01

**Authors:** Mohamad Mahdi Mortazavipour, Zeinab Mohamadalizadeh-Hanjani, Loabat Geranpayeh, Reza Mahdian, Shirin Shahbazi

**Affiliations:** 1 Department of Medical Genetics, Faculty of Medical Sciences, Tarbiat Modares University, ‎Tehran, Iran;; 2 Department of Surgery, Sina Hospital, Tehran University of Medical Sciences, Tehran, Iran;; 3 Molecular Medicine Department, Pasteur Institute of Iran, Tehran, Iran

## Abstract

**Background:**

K-Ras mutations rarely occur in breast cancer. However, studies have supported that K-Ras upregulation is involved in breast cancer pathogenesis. Two main K-Ras transcript variants, K-Ras4A and K-Ras4B, arise originate from the alternative splicing of exon 4. In this study, we aimed to evaluate variations in the expression of K-Ras4A and K-Ras4B and their role in breast ductal carcinoma.

**Methods:**

Total RNA was extracted from breast tumors, and the NATs were obtained via mastectomy. Patients were selected from new cases of breast cancer with no prior history of chemotherapy. Relative mRNA expression was calculated based on a pairwise comparison between the tumors and the NATs following normalization to the internal control gene. Predictive values of the transcript variants were examined by ROC curve analysis.

**Results:**

A statistically significant increase was found in K-Ras4A and K-Ras4B expression with the mean fold changes of 7.58 (*p *= 0.01) and 2.47 (*p *= 0.001), respectively. The K-Ras4A/K-Ras4B ratio was lower in the tumors than that of the normal tissues. ROC curve analysis revealed the potential of K-Ras4A (AUC: 0.769) and K-Ras4B (AUC: 0.688) in breast cancer prediction. There was also a significant association between K-Ras4B expression and HER2 statues (*p* = 0.04). Furthermore, a significant link was detected between K-Ras4A expression and pathological prognostic stages (*p* = 0.04).

**Conclusion:**

Our findings reveal that the expression levels of K-Ras4A and K-Ras4B is higher in the tumor compared to the normal breast tissues. Increase in K-Ras4A expression was more significant than that of K-Ras4B.

## INTRODUCTION

Despite recent developments in the diagnosis and treatment of human diseases, cancer is still the most prominent cause of mortality, worldwide. Iranian National Population-Based Cancer Registry has reported the age-standardized incidence rate of breast cancer as 34.53 per 100,000 and considered breast malignancies the most common cancer among Iranian women^[^^1^^]^. Histologically, breast cancer is divided into two main overarching groups: carcinomas and sarcomas^[^^2^^]^. Breast carcinomas start in the epithelial cells, and consist of either ductal or lobular types, depending on their origin^[^^3^^]^. About one-third of human cancers with epithelial origin have dysregulated Ras/MAP/ERK signaling pathway^[^^4^^]^. Following activation of the cell surface receptors by extracellular ligands, Ras GTPase triggers signal transducing, and subsequently cell growth, differentiation, and survival. The gain-of-function mutations of the Ras proto-oncogenes deregulates cancer signaling pathways, leading to tumorigenesis and metastasis^[^^5^^]^. K-Ras is the most clinically significant members of the Ras subfamily and frequently mutated gene contributing to tumorigenesis in human. Numerous mutations of K-Ras are detected in a variety of cancers, including colorectal, lung, and pancreatic, but rarely in breast cancer^[^^6^^,^^7^^]^.

K-Ras gene has two main transcript variants, known as K-Ras4A and K-Ras4B. Dissimilarity between these two isoform roots in the alternative splicing of exon 4, resulting in K-Ras4A with 189 amino acids vs. 188 residues in K-Ras4B^[^^8^^]^. Functionally, the key difference between K-Ras4A and K-Ras4B exists in their post-translation modifications. C-terminal hyper-variable regions are typically presented on all Ras proteins. It consists of a CAAX motif, which undergoes cysteine farnesylation and permits an amplified Ras localization to the plasma membrane. K-Ras4B has a charge-mediated polybasic sequence that is located in the upstream of the CAAX motif and contains multiple lysine residues^[^^9^^]^ by which K-Ras4B interacts with calmodulin. Since ductal tissues contain a high level of calcium and calmodulin, K-Ras4B could contribute to tumorigenesis by activating calmodulin/PI3Kα/Akt pathway^[^^10^^]^. K-Ras4A comprises polybasic region, as well as cysteine residue for palmitoylation modification, which also occurs in H-Ras and N-Ras^[^^11^^]^. K-Ras4A exists in both farnesylated/ nonpalmitoylated and farnesylated/ palmitoylated forms, contributing to two distinct signaling pathways as K-Ras4B and N-Ras, respectively. This characteristic of K-Ras4A explains its involvement in a broader range of cancers^[^^12^^]^. 

Herein, we aimed to study the expression of K-Ras4A and K-Ras4B and determination of their ratio in a series of ductal carcinoma of breast samples. Given the fact that K-Ras4A and K-Ras4B act differently in cancers, we intended to reveal which variant is the main transcript involving in breast tumor pathogenesis.

## MATERIALS AND METHODS

**Patients**

Breast cancer cases were selected from the patients referred to the Department of Surgery, Sina Hospital, Tehran, Iran. The volunteered individuals participated in the study based on the following inclusion criteria, i.e. new cases of breast ductal carcinoma confirmed by biopsy. Exclusion criteria were patients who received no chemotherapy, radiotherapy, and hormone therapy before surgery and sampling. The patients were examined by an expert surgeon and evaluated according to the standard imaging procedures. 

**Sample collection and verification**

Seventy tumors and the matched NATs were obtained via mastectomy and then sectioned into two replicates. One replicate was instantly immersed into liquid nitrogen containers for long-term storage, and the other one was examined by an expert pathologist. Presence of ER, PR, and HER2 was assessed by IHC assay. Envision method was applied as a two-step technique, based on the dextran polymer technology. The antibody against Ki67 nuclear antigen was used to determine the cell proportion of luminal tumor subtypes. The cut-off value of 14% was considered to define the Ki67 index. Since our study was conducted on the patients who performed surgery as the initial treatment, we assigned them into the pathologic prognostic stage (AJCC 2017, the 8^th^ edition). 

**RNA extraction and real-time PCR**

Total RNA was extracted from the tumors and NATs using GeneAll®RNA extraction kit (Pishgam, Iran) according to the manufacturer's instructions. RNA samples with high purity (OD_260/280_ >1.8) were used as the template for complementary DNA synthesis carried out by using BioFact™ RT Series kit (Noavaran Teb Beynolmelal, Iran). Quantitative real-time RT-PCR assay was set up by selecting *PUM1* gene as an internal control. K-Ras4A, K-Ras4B, and PUM1 specific primers indicated in Table 1, were applied as described before^[^^13^^,^^14^^]^. Amplification was performed by Biofact SYBR-Green High-ROX master mix (Noavaran Teb Beynolmelal) as follows: 95 °C for 15 min; 40 cycles at 95 °C for 20 s and 60 °C for 40 s. Real-time RT-PCR was performed in duplicates for each sample, as well as no template control. The reaction mixtures were run on StepOne Plus^®^ (Life Sciences, USA). The Ct values were obtained from the data output of the instrument software. The melting curves were created for each amplified fragment to determine the PCR specificity. Raw amplification data were transferred to LinReg software, and linear standard curves were generated.

**Table 1:** Specified primers designed for quantitative real-time PCR

Gene	Sequence (5`_3`)	Length	Tm	GC (%)	Product (bp)
*PUM1*	CCTACCAACTCATGGTGGATGT	22	59	50	83
	AGCCAGCTTCTGTTCAAGACT	21	59	47	
					
*K-Ras4A*	AAAGACAAGACAGAGAGTGGAG	22	57	45	151
	GCATCATCAACACCCAGATTAC	22	57	45	
					
*K-Ras4B*	TGAGGACTGGGGAGGGCTTT	20	62	60	252
	AGGCATCATCAACACCCTGTCT	22	61	50	

Tm, melting temperature; GC%, percentage of guanine and cytosine content

**Gene expression analysis**

The mean Ct values of K-Ras4A and K-Ras4B were normalized to that of PUM1, and ΔCt was determined for each sample. \the fold changes were then calculated based on a pairwise comparison between ΔCt of the tumors and the NATs of the same patient using the relative expression software tool (REST©2009, Qiagen, Germany). Gene expression variations with more than two-fold changes were considered significant. Fold change significance was shown by ggplot2 and ggsignif packages in R language environment. The pheatmap package was applied to visualize log2Fc against the selected pathological parameters. 

**ROC curve assessment**

Relative distribution of K-Ras4A and K-Ras4B expression was analyzed by ROC curve. pROC package in the R was used for plotting ΔCt of the transcripts. A logistic regression model was recruited to calculate the AUC and 95% CIs. Sensitivity and specificity were calculated to compare the predictive values of the transcripts by caret and lattice packages. 

Statistical analysis 

The sample size was calculated by EpI Info 7.0.9.34 software. To reject the null hypothesis of no K-Ras4A and K-Ras4B effects, the significance level was chosen at 5% to ensure a power of 80%. The collected clinical and pathological data were statistically analyzed using SPSS version 24. Correlation studies were performed by Chi-square tests.

## RESULTS

**Patients' clinicopathological characteristics**

**Fig. 1:**
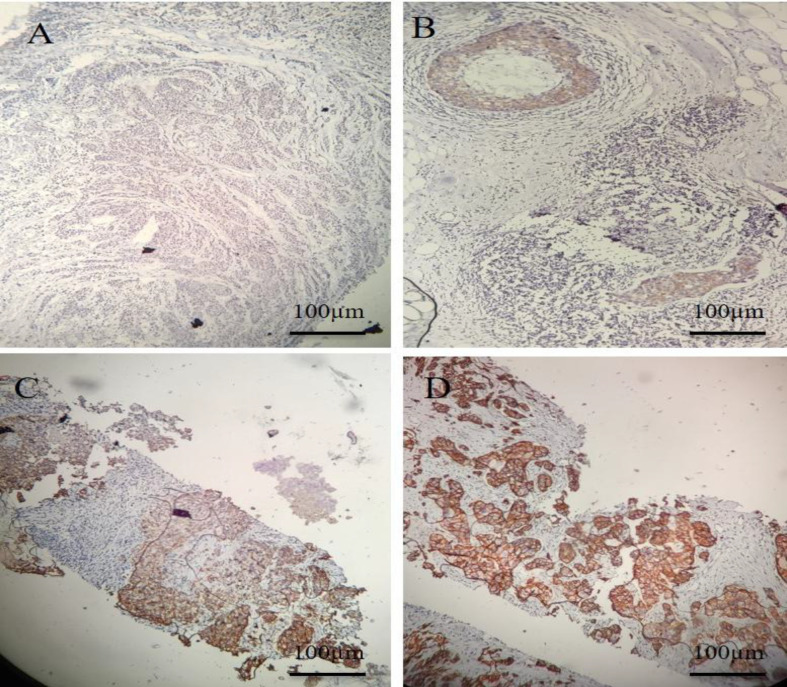
HER2 analysis by IHC was categorized into (A) negative, (B) positive+, (C) positive++, and (D) positive+++. Envision method was applied as a two-step technique, based on the dextran polymer technology

The mean age of patients was 56.52 ± 13.37 years old, ranging from 31 to 82. Thirty-five percentage of the patients had a positive family history of cancer among their first- or second-degree relatives (Table 2). Despite several affected family members, the pedigrees did not follow the Mendelian inheritance pattern in any of the families. HER2 status analysis by IHC was categorized into negative, positive+, positive++, and positive+++ (Fig. 1). The obtained results revealed that 83% of the tumors were HER2-negative. Based on the histopathological results, 17% and 23% of the tumors did not express ER and PR, respectively (Table 2). Molecular subtypes, including luminal A and B, HER2-positive, and triple-negative (basal-like) were assigned based on the ER, PR, HER2, and Ki67 expression in the tumor tissues. According to these findings, 34% of the tumor tissues were luminal A, and 48% was assigned to luminal B subgroup. HER2-positive and triple-negative comprised 6% and 12% of the tissues, respectively. The cases were also characterized based on the pathological prognostic stages according to the AJCC guideline. The stages were determined by considering tumor size, lymph node involvement, and metastasis in addition to tumor grade and status of ER, PR and HER2. Nearly two-third of the patients was classified into stage I, and the rest was almost equally distributed to stages II and III (Table 2). 

**K-Ras4B and K-Ras4A gene expression**

**Fig. 2:**
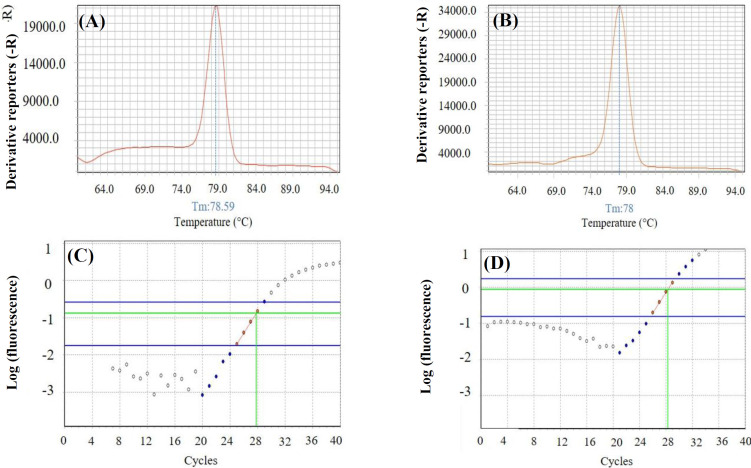
Melting curve of (A) 252-bp amplified fragment of K-Ras4B and (B) 151-bp amplified fragment of K-Ras4A; transferred amplification data of (C) K-Ras4B and (D) K-Ras4A to LinReg software and linear standard curves

**Fig. 3:**
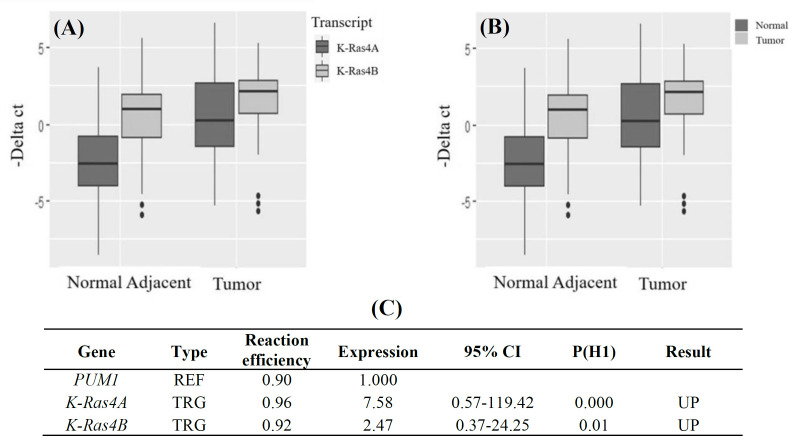
ΔCt value of (A) K-Ras4A and (B) K-Ras4B plot showing the normalized relative expressions of K-Ras4A as well as K-Ras4B analyzed by REST© software; (C) fold changes calculated with the internal control PUM1 and normalized to the expression of the respective genes in NATS. UP, upregulation

Efficiency of the primers was calculated by LinReg software to correct the confounding effects of possible differences (Fig. 2). ΔCt values obtained by normalization to the PUM1 Ct, are shown in Figure 3A. As indicated in Figure 2, expression of the variants was higher in the tumors than that of NATs. By comparing two transcripts, we observed that the expression level of K-Ras4B was higher than K-Ras4A in the tumors, as well as NATs, but a lower difference was detected in the expression level of K-Ras4B compared to K-Ras4A. The mean ΔCts of K-RasA were 0.38 and -2.55 in the tumor and NATs, respectively. The values of these expression levels were calculated as 1.77 and 0.47 for K-RasB in the tumor and NATs, respectively. The calculated mean fold changes by using REST^©^ software identified a significant increase in K-Ras4A and also K-Ras4B mRNA expression level in the breast tumors compared with NATs. The fold change of K-Ras4B was 2.47, with 95% CI = 0.37-24.25 and *p =* 0.001. Of note, the increase in the expression level was even more remarkable for K-Ras4A compared to K-Ras4B, displaying a fold change of 7.58, 95% CI = 0.57-119.42, and *p =* 0.01 (Fig. 3B and 3C).

**Table 2:** Correlation between the expression levels of K-Ras4A and K-Ras4B (fold changes) and clinicopathological parameters of breast tumor samples

**Clinical parameters**	**Number (%)**		**K-Ras4A expression**						**K-Ras4B expression**			
			**Low** **n = 7** **(20%)**	**High** **n = 28** **(80%)**	***p*** **value**		**χ2**		**Low** **n = 19** **(54%)**	**High** **n = 16** **(46%)**	***p*** **value**	**χ2**
**Age**												
<50	13 (37)		4	9	0.22		1.499		7	6	0.96	0.002
≥50	22 (63)		3	19					12	10		
								
**Family history**												
No	23 (65)		4	19	0.59		0.285		11	12	0.28	1.128
Yes	12 (35)		3	9					8	4		
								
**ER**												
Negative	6 (17)		2	4	0.37		0.805		4	2	0.50	0.447
Positive	29 (83)		5	24					15	14		
								
**PR**												
Negative	8 (23)		3	5	0.15	1.985			5	3	0.59	0.282
Positive	27 (77)		4	23					14	13		
								
**HER2**												
Negative	29 (83)		5	24	0.37		0.805		18	11	**0.04**	4.130
Positive	6 (17)		2	4					1	5		
								
**Ki67**												
>14%	12 (34)		1	11	0.21		1.553		6	6	0.71	0.135
<14%	23 (66)		6	17					13	10		
								
**Pathological prognostic stages**												
Stage I	26 (74)		4	22	**0.04**		6.346		13	13	0.62	0.950
Stage II	5 (14)		3	2					3	2		
Stage III	4 (12)		0	4					3	1		
								
**Molecular subtypes**												
Luminal A	12 (34)		1	11	0.50		2.341		6	6	0.84	0.807
Luminal B	17 (48)		4	13					9	8		
HER2-positive	2 (6)		1	1					1	1		
Basal-like	4 (12)		1	3					3	1		

Pathological prognostic stages were determined by the tumor size, lymph node involvement, and metastasis system in addition to tumor grade, ER, PR and HER2 status according to AJCC 8^th^ guideline. Chi-square was applied to compare the K-Ras4A and K-Ras4B expression levels according to the clinicopathological features. The bold numbers show statistical significance.

**Differential detection power of K-Ras transcript variants**

**Fig. 4:**
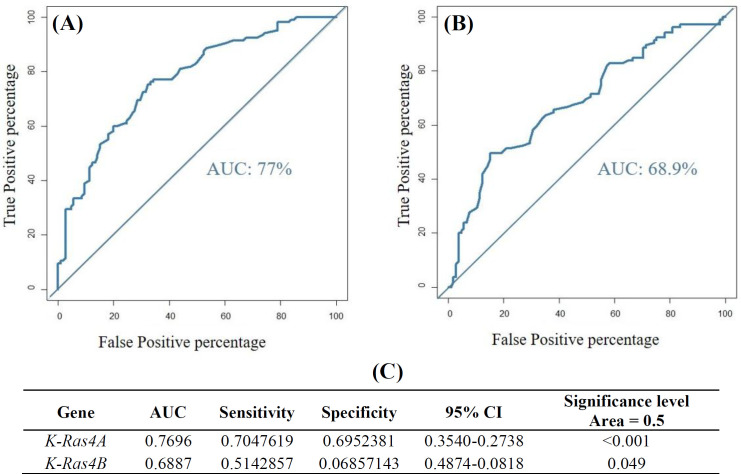
ROC curve analysis evaluating the performance of (A) K-Ras4A and (B) K-Ras4B; (C) statistical analysis results of logistic regression model presenting AUC and 95% CI. Sensitivity and specificity were calculated to compare the predictive values of the transcripts

ROC curve analysis was applied to evaluate the rate of false-positive prediction. As depicted in Figure 4, the results of ROC curve showed an AUC of 0.769, sensitivity of 70.47, and specificity of 69.52 for K-Ras4A. Meanwhile, K-Ras4B displayed AUC of 0.688, sensitivity of 51.42, and specificity of 68.57, indicating a more significant reliability of K-Ras4A than that of K-Ras4B in breast cancer prediction. 

**Correlation between** **expression and clinicopathological status of K-Ras variants **

The significance of the K-Ras4A and K-Ras4B expression was determined based on the clinicopathological features of the patients. The correlations were studied using the Chi-square test, and the values lower than 0.05 were considered significant. Samples were classified into two groups: high expression (fold change >2) and low expression (fold change ≤2) levels. K-Ras4B expression was significantly associated with HER2 status (*p* = 0.04).

**Fig. 5:**
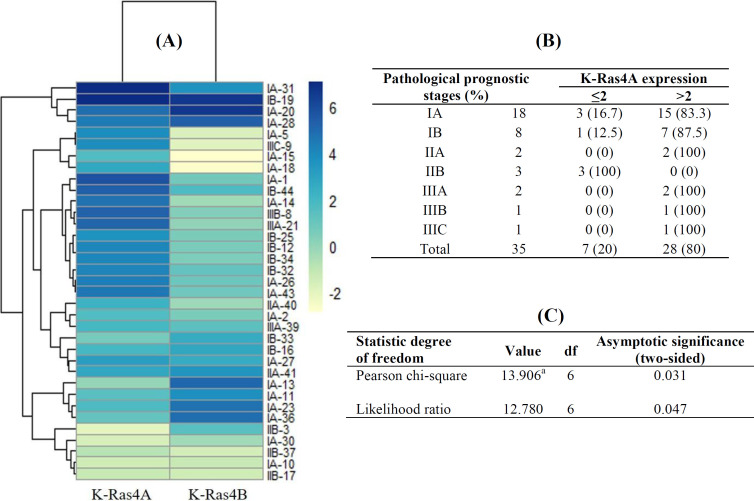
(A) Heatmap showing the correlation between log2Fc of K-Ras4A as well as K-Ras4B and pathological prognostic stages; (B) and (C) details of the statistical analysis are given in tables

However, results exhibited no significant difference between molecular subtypes, with K-Ras4B expression (Table 2). Statistical analysis also revealed a significant link between K-Ras4A expression and pathological prognostic stages (*p* = 0.04; Table 2). Figure 5 shows the log2Fc heatmap of K-Ras4A and K-Ras4B in each sample related to the pathological prognostic stages. This outcome indicated that the expression profile of K-Ras4A in breast tumors is distinct in different stages. Statistical correlation studies remained significant when the sub-stages were analyzed related to K-Ras4A (*p* = 0.03; Fig. 5). 

## DISCUSSION

In this study, we examined the expression profile of K-Ras4A and K-Ras4B in ductal carcinoma of breast. The results showed a significant increase in both transcripts in favor of K-Ras4A, although the level of K-Ras4B was higher than that of K-Ras4A in both tumors and NATs. Of note, increase of K-Ras4A in the tumors was far more significant compared to NATs,. Generally, K-Ras4B was considered as the main K-Ras transcript, and the reports relating to K-Ras4A expression were limited to a few numbers of tissues. Contrary to primary studies, Tsai and collogues^[^^15^^]^ showed that K-Ras4A is expressed in a wide range of cancer cell lines. In a majority of the cell lines, K-Ras4A represents about one-quarter of the total K-Ras transcripts, which increased by half in the colorectal tumors. In breast cancer cell lines, MCF-7, MDA-MD-231, and MDA-MD-468, K-Ras4A is accounted for 25% of the total K-Ras^[^^15^^]^. Studies on the advanced non-small-cell lung cancer not only reported the higher levels of K-Ras4B than K-Ras4A, but also showed a significant more up-regulation of K-Ras4A in the tumor compared to the normal tissues, which are in agreement with our results^[^^16^^]^. 

**Fig. 6:**
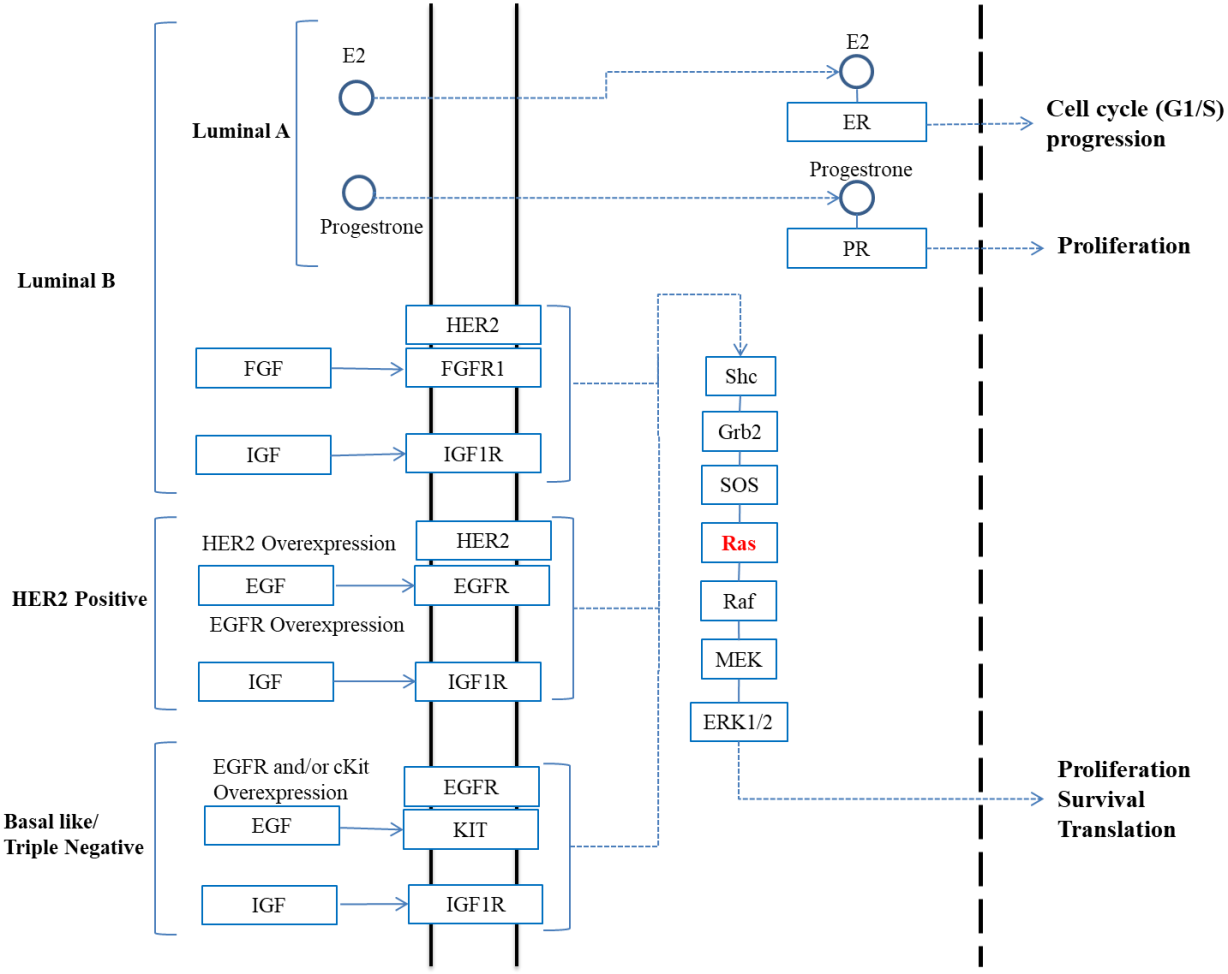
Involvement of Ras and estrogen/progesterone pathways in cell proliferation of breast cancer molecular subtypes (summarized from KEGG)

In our previous study on endometriosis, we found an increased expression of K-Ras4A in different phases of menstruation, influenced by estrogen and/or progesterone levels^[^^17^^]^. However, in the present study, we did not find any link between hormonal status of breast tumor and K-Ras4A or K-Ras4B expression. This finding might be due to divergence in the functional pathways of RAS and estrogen/progesterone in breast tumors. Figure 6 summarizes the cell proliferation pathways in breast cancer adapted from the KEGG database (https://www.genome.jp/kegg-bin/show_pathway?hsa0 5224+3845). As indicated in Figure 6, in luminal A, the signals are mainly transmited via the hormone receptors. However, the RAS/Raf/MEK signaling pathway is a key cell proliferation path in luminal B, HER2, and basal-like subtypes. This Figure 6 also shows that HER2 activity can enhance the RAS/Raf/MEK path signaling. In this regard, our results exhibited a significant correlation between HER2-positive tumors and K-Ras4B, but not K-Ras4A expression. 

We observed a significant link between K-Ras4A and newly developed AJCC pathological prognostic stages, which accurately predict the survival outcomes compared to the anatomical stages^[^^18^^]^. Various studies have been reported the role of K-Ras transcripts in cancer;however, the results are still contradictory. In most publications, K-Ras4B was found to have an anti-apoptotic function^[^^19^^]^. However, in a mouse lung cancer model, K-Ras4B reduced tumor number and size and it was regarded as an inhibitor of tumor progression^[^^19^^]^. K-Ras4A also has a tumor suppressor role as well as proapoptotic effects demonstrating in the K-Ras4A knockout mice, which corroborates a disrupted apoptosis process^[^^20^^]^. Furthermore, induction of colonic adenomas in K-Ras4A knockout mice resulted in more and larger tumors, confirming that the K-Ras4A acts as a tumor suppressor^[^^20^^]^. Other studies have also drawn attention to the expression ratio of these transcript variants, i.e. K-Ras4A and K-Ras4B. In this context, Luo and colleagues^[^^21^^]^ observed an increase in the expression level of K-Ras4B in mice with homozygous targeted deletions of K-ras exon 4A. They concluded that the main effect of K-Ras isoforms on apoptosis and/or proliferation, depends on the K-Ras4A/K-Ras4B ratio. In our study, K-Ras4A/K-Ras4B ratio increased in the tumor tissues. Our results showed no correlation with a previous investigation performed on human colon cancer cell lines and colorectal tumors and reported a significant reduction in K-Ras4A/K-Ras4B ratio^[^^22^^]^. Moreover, a role for epigenetic and histone modification has recently been revealed, which affects the K-Ras4A/K-Ras4B ratio in colorectal cancer cell lines^[^^23^^]^. It should be considered that in colon cancer, K-Ras transcripts mostly bear mutations, while the K-Ras mutations are rare in breast cancer. 

Altogether, it is worthwhile to further investigate the role of K-Ras4A in breast cancer prognosis. Based on the TCGA and METABRIC databases, K-Ras mRNA expression has been reported as an independent prognostic factor for luminal A breast cancer subtype^[^^24^^]^. Given that the RAS/Raf/MEK does not play a significant role in the luminal A, it is interesting to study the transcript variants, separately. A recent study has also identified the K-Ras gene expression as a prognostic factor associating with tumor immune infiltration in breast cancer. However, they did not report a clear difference in the K-Ras gene expression between the molecular subtypes^[^^25^^]^.

K-Ras proto-oncogene is one of the important candidates in cancer therapy. Elucidating the role of K-Ras4B and K-Ras4A in breast cancer leads to a coherent understanding of the crucial transcript as a therapeutic target. The findings of our study show that K-Ras4A expression level substantially increases in breast cancer, indicating its key role in the disease pathogenesis. 

## DECLARATIONS

### Acknowledgments

The author would like to thank the patients who participated in this survey.

### Ethical statement

The study protocol was approved by the Ethics Committee of Tarbiat Modares University, Tehran, Iran (ethical code: IR.TMU.REC.1396.681). Each patient signed a written informed consent. All the authors have read and approved the contents of the final manuscript and agreed to publicize this manuscript

### Data availability

The raw data supporting the conclusions of this article are available from the authors upon reasonable request. 

### Author contributions

MMM: project development, data collection, and manuscript writing/editing. ZMH: data collection and manuscript editing; LG: data collection and management, and manuscript editing; RM: project development, data analysis, and manuscript editing. SS: project development, data collection and management, data analysis, and manuscript writing/ editing. 

### Conflict of interest

The authors declare no competing interests.

### Funding/support

This study has received no financial support from any agency in the public, commercial, or nonprofit sectors.
